# Coats plus syndrome with new observation of drusenoid retinal pigment epithelial detachments in a teenager^☆^

**DOI:** 10.1016/j.ajoc.2022.101713

**Published:** 2022-09-21

**Authors:** Kushal U. Agrawal, Nicholas E. Kalafatis, Carol L. Shields

**Affiliations:** Ocular Oncology Service, Wills Eye Hospital, Thomas Jefferson University, 840 Walnut Street, 14th Floor, Philadelphia, PA, 19107, USA

**Keywords:** Telomere biology disorder, Coats plus syndrome, Dyskeratosis congenita, CTC1 gene, Pigment epithelial detachment

## Abstract

**Purpose:**

To describe a case of Coats Plus Syndrome (CPS), a vision and life threatening disease belonging to a family of diseases known as the Telomere Biology Disorders.

**Observations:**

A 15-year-old girl with a history of small for gestational age, short stature, microcephaly, thinning/greying of scalp hair, skin hyperpigmentation, nail ridging, and multiple pathological fractures presented with bilateral Coats-like retinopathy. We discovered a new observation of multiple peripheral pinpoint retinal pigment epithelial detachments (PEDs). Further genetic testing revealed CTC1 gene mutation and she was diagnosed with Coats plus syndrome with features of dyskeratosis congenita, a telomere biology disorder.

**Conclusion and importance:**

Patients with bilateral Coats-like retinopathy and associated systemic features suggestive of CPS should be evaluated through genetic testing to diagnose this disease and treat vision and life threatening manifestations as early as possible. In this report, we also document, for the first time, multiple pinpoint PEDs that could be related to an accelerated aging process with telomere dysfunction.

## 1 Introduction

Telomere biology disorders (TBD) are group of genetic diseases associated with several systemic and ocular manifestations.^1^ In some patients, ocular manifestations can be the first presenting signs, and knowledge of systemic associations of TBD is critical for ophthalmologists to diagnose this disorder early. Coats Plus Syndrome (CPS) is one of the TBDs associated with bilateral Coats-like retinopathy. In this report, we describe a case of CPS and its systemic associations to highlight key features that should raise suspicion for this disease and prompt genetic testing.

## 2 Case report

A 15-year-old Caucasian female with a history of small for gestational age at birth and multiple atraumatic pathological bone fractures of the femur and humerus over three years was referred for ophthalmic examination.

**Fig. 1:**
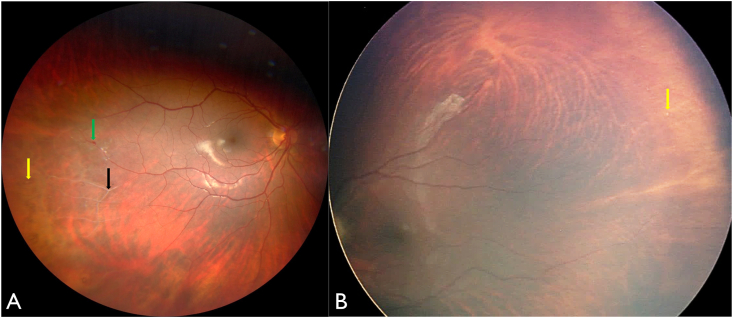
(A) Fundus photograph of the right eye demonstrating sclerosed retinal vessels (black arrow), telangiectasia (green arrow), and drusen-like deposits (yellow arrow). (B) Fundus photograph of the left eye showing similar drusen-like deposits (yellow arrow). (For interpretation of the references to colour in this figure legend, the reader is referred to the Web version of this article.)

**Fig. 2:**
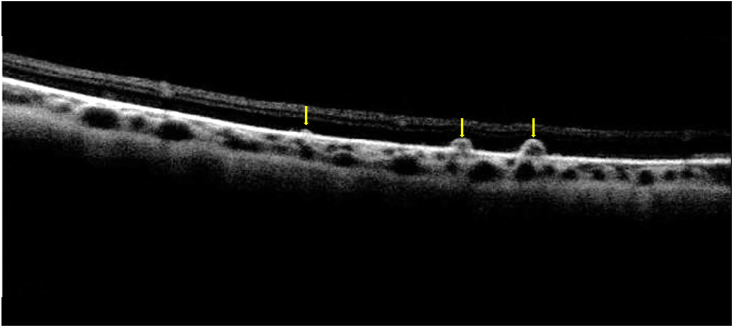
Optical coherence tomography (OCT) of the drusen-like deposits showing drusenoid retinal pigment epithelial (RPE) detachments (yellow arrows). (For interpretation of the references to colour in this figure legend, the reader is referred to the Web version of this article.)

**Fig. 3:**
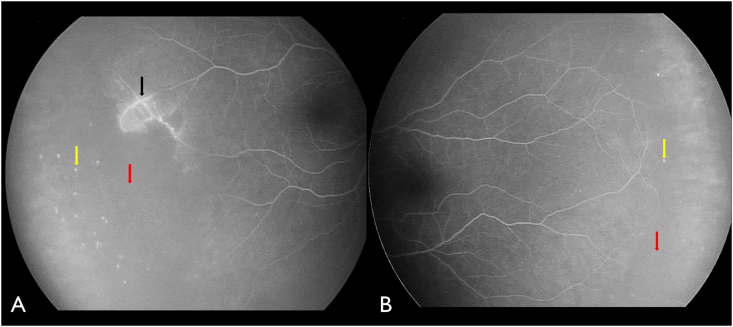
(A) Fluorescein angiography of the right eye showing minor telangiectasia, minimal leakage along the temporal blood vessels (black arrow) and broad peripheral nonperfusion (red arrow). Pinpoint staining in the peripheral drusenoid retinal pigment epithelial (RPE) detachments (yellow arrow) is noted. (B) The left eye showed minimal nonperfusion without leakage temporally (red arrow) and similar pinpoint staining of the RPE detachments (yellow arrow). (For interpretation of the references to colour in this figure legend, the reader is referred to the Web version of this article.)

On presentation, visual acuity was 20/30 in the right eye (OD) and 20/25 in the left eye (OS). Anterior segment examination was normal in both eyes (OU). Fundoscopy OD revealed retinal telangiectasia, subtle exudation inferior to telangiectatic vessel, and vascular sclerosis temporally, whereas fundoscopy OS showed clinically normal vascularity (Fig. 1). Both eyes demonstrated pinpoint round, drusen-like sub-retinal pigment epithelial (sub-RPE) deposits in the periphery, and on optical coherence tomography (OCT) these lesions were confirmed to be drusenoid retinal pigment epithelial detachments (PEDs) (Fig. 2). Fluorescein angiography (FA) revealed localized temporal non-perfusion with related telangiectasia and minimal leakage OD, minimal temporal non-perfusion without leakage OS, and pinpoint staining in the PEDs OU. (Fig. 3).

**Fig. 4:**
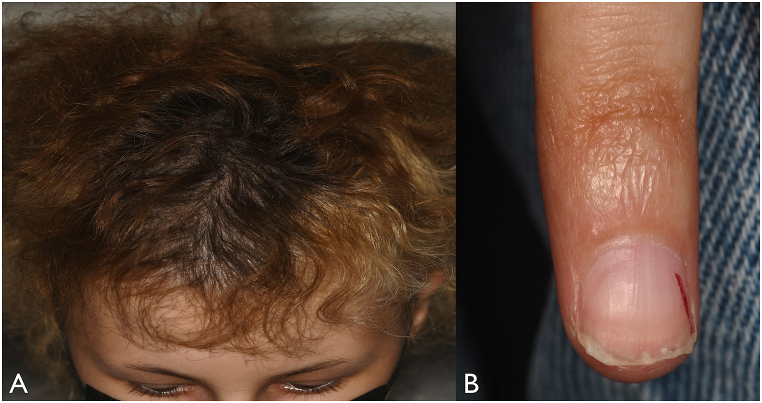
External photographs demonstrating (A) thin, premature greying of scalp hair and microcephaly and (B) ridged, dysplastic fingernail (remnant red fingernail polish laterally). (For interpretation of the references to colour in this figure legend, the reader is referred to the Web version of this article.)

**Fig. 5:**
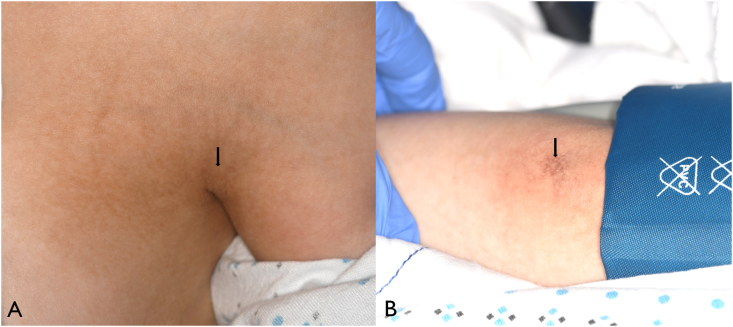
External photographs showing cutaneous hyperpigmentation in the (A) axilla (black arrow) and (B) antecubital fossa (black arrow).

On systemic examination, the 15-year-old female patient demonstrated short stature, microcephaly, sparse and premature greying of scalp hair, premature aging of skin, cutaneous hyperpigmentation of neck, axilla and elbow, neck eczema, ridged fingernails, and syndactyly of both feet (Fig. 4, Fig. 5). The ophthalmic features of bilateral retinal non-perfusion with exudative retinopathy, suggestive of Coats disease. Multiple systemic abnormalities raised suspicion for Coats plus syndrome (CPS) with features of dyskeratosis congenita. Whole Exome Sequencing revealed biallelic CTC1 gene (NM_025099.6:c.3514+3A > G) mutation (Division of Genomic Diagnostics at Children's Hospital Of Philadelphia, Philadelphia, PA, USA) with normal telomere length (thus far), confirming the diagnosis of CPS. The biallelic inheritance of a pathogenic variant of the CTC1 gene: c.277C > T, p.Gln93* was considered likely pathogenic and was paternally inherited, whereas c.2959C > T, p.Arg987Trp was considered known pathogenic and was maternally inherited.

The right eye was treated with laser photocoagulation to the areas of nonperfusion and leakage, and the left eye was observed. Magnetic resonance imaging (MRI) of the brain was normal without calcification or cyst. The dual-energy X-ray absorptiometry (DEXA) scan and complete blood count (CBC) were normal, suggesting lack of osteoporosis or bone marrow disorder. Bone marrow biopsy to screen for cellularity, dysplasia, cytogenetic and molecular abnormality is pending.

The final diagnosis was CPS in a teenager with features of dyskeratosis congenita and a new observation of bilateral drusenoid PEDs. Continuing ophthalmic and systemic evaluation for related conditions was advised.

## 3 Discussion

Coats plus syndrome represents one condition on the spectrum of the rare telomere biology disorders (TBD), a family of disorders identified by genetic mutations affecting telomere health.^1^ Telomeres are repetitive nucleotides at the ends of chromosomes that are important for DNA stability and protection.^2^ During each cycle of cell division, tiny fragments of the telomeres are lost, serving as a “scapegoat” to prevent DNA damage.^2^^,^^3^ The pathophysiology of TBDs stems from a dysfunction in telomerase, an enzyme responsible for maintenance and replenishment of telomeres.^2^ Dyskeratosis congenita, Hoyeraal-Hreidarsson syndrome, Revesz syndrome, and Coats plus syndrome are multisystemic TBDs while pulmonary fibrosis, aplastic anemia or liver disease are isolated TBDs.^2^

Dyskeratosis congenita (DC) is a well-described TBD characterized by a classic mucocutaneous triad including fingernail/toenail dysplasia, reticular skin pigmentation, and oral leukoplakia.^1^ Bone marrow failure is the most common cause of mortality in DC-affected individuals, followed by pulmonary disease and malignancy.^2^ The ophthalmic features of DC include bilateral peripheral vaso-occlusive and exudative retinopathy, nerve fiber layer infarction, macular edema, and optic atrophy.^4^

Coats plus syndrome is considered a more severe variant of DC.^1^ The genetic inheritance of CPS has been linked to a mutation in the CTC1 gene on chromosome 17p13.1 that encodes for conserved telomere maintenance component 1 (CTC1), which is responsible for maintaining telomeric structural integrity.^3^ CTC1 gene was reported to be associated with CPS by Anderson et al. from 10 families with CPS.^3^ This mutation most often occurs sporadically, although some reports suggest an autosomal-recessive pattern.^5^ There is a molecular link between DC and CPS, with overlapping genetic mutations, including CTC1, and clinical features.^2^

Other TBDs such as Hoyeraal-Heidrasson syndrome (features of DC plus cerebellar hypoplasia and immunodeficiency) and Revesz syndrome (features of DC plus bilateral exudative retinopathy) are also considered severe variants of DC.^1^ The overlapping features of Revesz syndrome and CPS include bilateral exudative retinopathy, and the differentiating features are TINF2 mutation with autosomal dominant inheritance in Revesz syndrome versus CTC1 mutation, osteopenia, fracture predisposition and recurrent gastrointestinal hemorrhages in CPS.^1^

Typical Coats disease is an idiopathic, unilateral retinal vascular disorder with telangiectasia and intraretinal and/or subretinal exudation without appreciable retinal or vitreal traction.^6^ A prominent feature on fluorescein angiography in Coats disease is peripheral non-perfusion, typically inferotemporally.^7^ Treatment with laser photocoagulation or cryotherapy to the telangiectasia and sometimes to the nonperfusion can lead to resolution of exudative retinopathy. In advanced disease, total exudative retinal detachment may require external drainage and subsequent treatment of the telangiectasia. Those with secondary glaucoma or phthisis bulbi often require enucleation.^6^

Coats plus syndrome is a rare condition characterized by bilateral “Coats-like” retinopathy plus intrauterine growth restriction (IUGR), short stature, osteopenia, and other systemic disorders.^3^^,^^7^^,^^8^ Reported retinal manifestations of coats plus syndrome can range from capillary telangiectasias, exudation with avascularity of peripheral retina, vascular malformations and aneurysmal dilatations, neovascularisation, macular edema and exudative retinal detachment, but retinopathy is almost always bilateral.^9^ Both Coats disease and CPS demonstrate retinal non-perfusion with telangiectasia and leakage on fluorescein angiography (FA).^6^ It is particularly important to investigate both eyes on FA, as a clinically normal retina can have subclinical areas of nonperfusion, seen only on FA, as in this case, and can be instrumental in the diagnosis of CPS.

An additional new finding in our case of peripheral, bilateral pinpoint drusenoid PEDs has not been previously recorded in cases of CPS. These lesions were tiny and in the far periphery, and thus could be easily overlooked. Drusenoid PEDs are highly uncommon in young patients and typically found in older patients due to a drop in hydraulic conductivity of bruch's membrane with age leading to disruption of transport between retinal pigment epithelium and choroid.^10^ We speculate that this feature could be related to accelerated RPE aging associated with dysfunctional telomeres.

The systemic complications of CPS are numerous. Patients with CPS and other TBDs are at risk for life-threatening gastrointestinal bleeding from vascular ectasia in the stomach, small intestine and liver, aplastic anemia from bone marrow failure, pulmonary fibrosis, and liver disease.^2^^,^^3^ Additionally, intracranial calcification, leukodystrophy, brain cysts, microcephaly, developmental delay and psychiatric disorders can also occur.^2^^,^^3^ Affected patients are at risk for malignancies including acute myeloid leukemia, non-Hodgkin lymphoma, head and neck squamous cell carcinoma, non-melanoma skin cancer, tongue leukoplakia, and others.^2^ Systemic surveillance is important with the TBDs.

Han et al. reviewed a 9 month old with CPS, with IUGR and anemia as well as bilateral Coats disease. Subsequent CTC1 gene analysis at 7 years of age was positive.^9^ Thus, manifestations of associated diseases with CPS and DC are progressive over time and require a high degree of suspicion for early detection.

## 4 Conclusion

In summary, we describe a 15-year-old girl with bilateral Coats-like retinopathy as well as drusenoid PEDs, small stature, slow growth, microcephaly, premature aging, and skin lesions, suggestive of telomere biology abnormality. Future evaluation for bone marrow stability is critical in this condition. Patients with bilateral Coats-like retinopathy with associated systemic abnormalities suggestive of CPS should be evaluated through genetic testing to diagnose and treat this disease early. We also document for the first time, multiple pinpoint PEDs and we hypothesize that it could be related to accelerated aging process of TBD.

## Patient consent

Consent to publish this case report has been obtained from the patient in writing at the Wills Eye Hospital, Ocular Oncology Service.

## Funding

No funding or grant support.

## Authorship

All authors attest that they meet the current ICMJE criteria for authorship.

## Declaration of competing interest

The authors have no disclosures to report.
